# HIV Infection of Naturally Occurring and Genetically Reprogrammed Human Regulatory T-cells

**DOI:** 10.1371/journal.pbio.0020198

**Published:** 2004-07-13

**Authors:** Kyra Oswald-Richter, Stacy M Grill, Nikki Shariat, Mindy Leelawong, Mark S Sundrud, David W Haas, Derya Unutmaz

**Affiliations:** **1**Department of Microbiology and Immunology, Vanderbilt University Medical SchoolNashville, Tennessee, United States of America; **2**Department of Medicine, Vanderbilt University Medical SchoolNashville, TennesseeUnited States of America

## Abstract

A T-cell subset, defined as CD4^+^CD25^hi^ (regulatory T-cells [Treg cells]), was recently shown to suppress T-cell activation. We demonstrate that human Treg cells isolated from healthy donors express the HIV-coreceptor CCR5 and are highly susceptible to HIV infection and replication. Because Treg cells are present in very few numbers and are difficult to expand in vitro, we genetically modified conventional human T-cells to generate Treg cells in vitro by ectopic expression of FoxP3, a transcription factor associated with reprogramming T-cells into a Treg subset. Overexpression of FoxP3 in naïve human CD4^+^ T-cells recapitulated the hyporesponsiveness and suppressive function of naturally occurring Treg cells. However, FoxP3 was less efficient in reprogramming memory T-cell subset into regulatory cells. In addition, FoxP3-transduced T-cells also became more susceptible to HIV infection. Remarkably, a portion of HIV-positive individuals with a low percentage of CD4^+^ and higher levels of activated T-cells have greatly reduced levels of FoxP3^+^CD4^+^CD25^hi^ T-cells, suggesting disruption of the Treg cells during HIV infection. Targeting and disruption of the T-cell regulatory system by HIV may contribute to hyperactivation of conventional T-cells, a characteristic of HIV disease progression. Moreover, the ability to reprogram human T-cells into Treg cells in vitro will greatly aid in decoding their mechanism of suppression, their enhanced susceptibility to HIV infection, and the unique markers expressed by this subset.

## Abstract 2

Human regulatory T-cells cells (Treg) -- or conventional T cells that have been genetically modified to generate Tregs -- express the HIV-coreceptor CCR5 and are susceptible to HIV infection

## Introduction

There is now compelling evidence that a subset of T-cells with regulatory activity suppresses T-cell activation in both mice and humans (Sakaguchi et al. 1995; Asano et al. 1996; Suri-Payer et al. 1998; Takahashi et al. 1998; Thornton and Shevach 1998; Baecher-Allan et al. 2001; Dieckmann et al. 2001; Jonuleit et al. 2001, 2002; Levings et al. 2001; Ng et al. 2001; Taams et al. 2001). Regulatory T-cells (Treg cells) have been shown to inhibit various autoimmune and allergic diseases (Shevach 2000; Furtado et al. 2001; Curotto de Lafaille and Lafaille 2002; Green et al. 2002, 2003; McHugh and Shevach 2002), mediate transplantation and self-tolerance (Sakaguchi et al. 1995; Hara et al. 2001; Taylor et al. 2001, 2002; Sanchez-Fueyo et al. 2002), and block the activation and proliferation of T-cells both in vitro and in vivo (Takahashi et al. 1998; Thornton and Shevach 1998; Annacker et al. 2000, 2001). These findings strongly suggest that Treg cells play a key role in immune regulation.

Human and murine Treg cells are functionally characterized by a decrease in both proliferation and IL-2 secretion in response to T-cell receptor (TCR) stimulation and by their ability to suppress activation of conventional T-cells (Asano et al. 1996; Takahashi et al. 1998; Thornton and Shevach 1998; Baecher-Allan et al. 2001; Dieckmann et al. 2001; Jonuleit et al. 2001; Levings et al. 2001; Ng et al. 2001; Taams et al. 2001, 2002). Treg cells mediate their suppressive effects only when stimulated via their TCRs (Takahashi et al. 1998; Thornton and Shevach 1998), although their suppressive effector function is antigen nonspecific (Thornton and Shevach 2000). Treg cells are clearly enriched within peripheral CD4^+^ T-cells that also express the α subunit of the IL-2 receptor (CD25), which is currently the best marker for identifying these cells (Shevach 2002). However, CD25 is also expressed on activated effector T-cells, and not all CD4^+^ Treg cells express CD25 (Annacker et al. 2001; Stephens et al. 2001). In adults, Treg cells are exclusively found in the CD45RO^+^ memory subset, and a sizable portion of these cells express the activation marker HLA-DR and the recently identified molecule glucocorticoid-induced tumor necrosis factor receptor (GITR, also known as TNFRSF18) (Gumperz et al. 2002; Lee et al. 2002). Upon activation, Treg cells express the inhibitory receptor CTLA-4 at a higher level and for a longer period of time than conventional T-cells (Read et al. 2000; Salomon et al. 2000; Takahashi et al. 2000). Interestingly, Treg cells have also been shown to express high levels of certain chemokine receptors such as CCR4 and CCR8 (Iellem et al. 2001).

The forkhead transcription factor FOXP3 was recently shown to be specifically expressed in mouse Treg cells and is required for their development (O'Garra and Vieira 2003; Ramsdell 2003). A mutation in the *FOXP3* gene carried by the *scurfy* mouse strain or a knockout of this gene causes a CD4^+^ T-cell-mediated lymphoproliferative disease characterized by cachexia and multiorgan lymphocytic infiltrates (Lyon et al. 1990; Brunkow et al. 2001). The human genetic disease immune dysregulation, polyendocrinopathy, enteropathy, X-linked syndrome (also called X-linked autoimmunity-allergic disregulation syndrome) is caused by mutations in the human homolog of *FoxP3* and is characterized by hyperactivation of T-cells with autoimmune endocrinopathy, early-onset type 1 diabetes and thyroiditis, and in some cases manifestations of severe atopy (Chatila et al. 2000; Bennett and Ochs 2001; Bennett et al. 2001; Wildin et al. 2001; Gambineri et al. 2003). In addition, expression of FOXP3 in conventional T-cells either in transgenic mice or by retroviral transduction is sufficient to confer a Treg cell phenotype (Fontenot et al. 2003; Hori et al. 2003; Khattri et al. 2003). However, the role of FoxP3 in the development of human Treg cells has not been examined.

The role of Treg cells in controlling T-cell activation during immune responses to pathogens such as chronic viral infections is currently a subject of great interest. Recently it was shown that Treg cells can regulate virus-specific or memory CD8^+^ T-cell responses, thus diminishing the magnitude of the immune response (Kursar et al. 2002, 2004; Murakami et al. 2002; Suvas et al. 2003; Aandahl et al. 2004). Because Treg cells express CD4**,** they are also potential targets of HIV in vivo. HIV entry into target cells also requires cellular expression of the chemokine receptors CCR5 or CXCR4 in conjunction with CD4 (Boshoff et al. 1997). However, the ability of HIV to establish a persistent infection is also critically dependent on activation signals that regulate HIV replication within target T-cells. Quiescent T-cells are resistant to infection unless TCR or cytokine activation signals are provided (Unutmaz 2001). Indeed, chronic states of T-cell hyperactivation, viral persistence, and T-cell depletion are all hallmarks of HIV infection (Grossman et al. 2002). Consequently, this state of chronic immune activation combined with the direct destruction of CD4^+^ T-cells by HIV leads to a profound immunodeficiency characterized by progressive deterioration of immune function (Fauci 1993). If Treg cells are lost because of HIV infection, this could potentially result in hyperactivation of conventional T-cells due to lack of immunoregulation. In contrast, if Treg cells are activated to expand during certain stages of the infection, this could have a suppressive effect on protective immune responses against the virus. Thus, in both scenarios dysregulation of Treg subset during HIV infection could have a profound impact on anti-HIV immune responses and pathogenesis of the infection.

We tested the susceptibility of both naturally occurring and in vitro genetically reprogrammed Treg cells to HIV infection. We found that Treg cells isolated from healthy donors express CCR5 and are highly susceptible to HIV infection. Ectopic expression of FoxP3 in conventional human T-cells genetically reprogrammed them into a Treg phenotype and enhanced their susceptibility to HIV infection. Remarkably, we also found a profound defect of FoxP3^+^CD4^+^CD25^hi^ T-cells in HIV-infected patients with low CD4^+^ and a high percentage of activated T-cells.

Our findings have important implications in understanding the role of Treg cells and the chronic activated state of T-cells during HIV infection. Furthermore, reprogramming of T-cells in vitro into Treg cells establishes a novel system to understand the mechanism of T-cell suppression and enhanced susceptibility of this subset to HIV infection.

## Results

### Isolation and Characterization of Human Treg Cells

**Figure 1:**
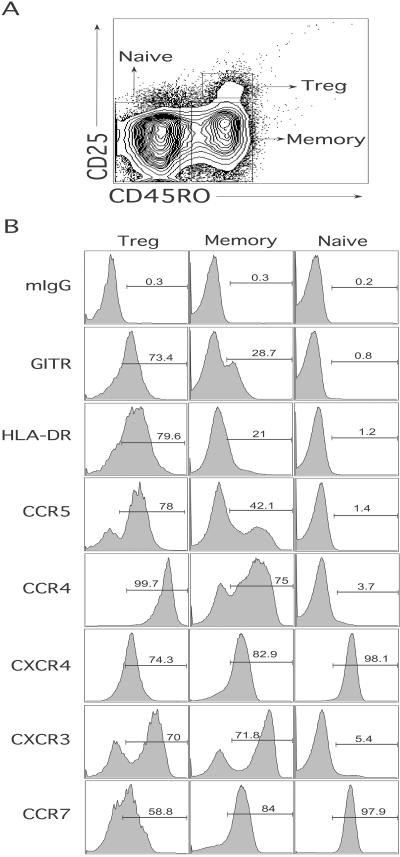
Identification and Phenotype of Treg Cells (A) Purified CD4^+^ T-cells were stained with anti-CD45RO-FITC and anti-CD25-PE antibodies. The naïve, memory, and Treg subsets were identified as shown in boxes. (B) Purified CD4^+^ T-cells were first stained with a pure antibody against the cell surface molecule shown in the figure, followed by antimouse IgG conjugated with allophycocyanin, followed by CD25-PE and CD45RO-FITC. Gates were set for Treg, memory, and naïve T-cells as shown in (A). These results are representative of one out of five donors analyzed.

To analyze susceptibility of Treg cells to HIV infection, we first developed a method to isolate these cells from peripheral blood. A sizable portion of human CD4^+^ T-cells (between 10%–20%) express CD25 (Figure 1A). However, approximately 1%–2% of CD4^+^ T-cells within the memory subset (CD45RO^+^) express high levels of CD25 (CD25^hi^) (Figure 1A). Previous studies suggested that human Treg cells resided within the CD45RO^+^CD25^hi^ subset (Baecher-Allan et al. 2001; Taams et al. 2001). We first performed a phenotypic analysis of CD45RO^+^CD25^hi^ (referred to as Treg), CD45RO^+^CD25^low/neg^ (memory T) and CD45RO^−^CD25^neg^ (naïve T) cells. Treg cells expressed higher levels of GITR and HLA-DR (Figure 1B), consistent with previous reports (Baecher-Allan et al. 2001; McHugh et al. 2002; Shimizu et al. 2002). Treg cells also expressed high levels of CCR5 and CCR4 compared to memory and naïve T-cells, while expression of CXCR4 and CCR7 was lower and CXCR3 expression was similar as compared to memory T-cells (Figure 1B).

**Figure 2:**
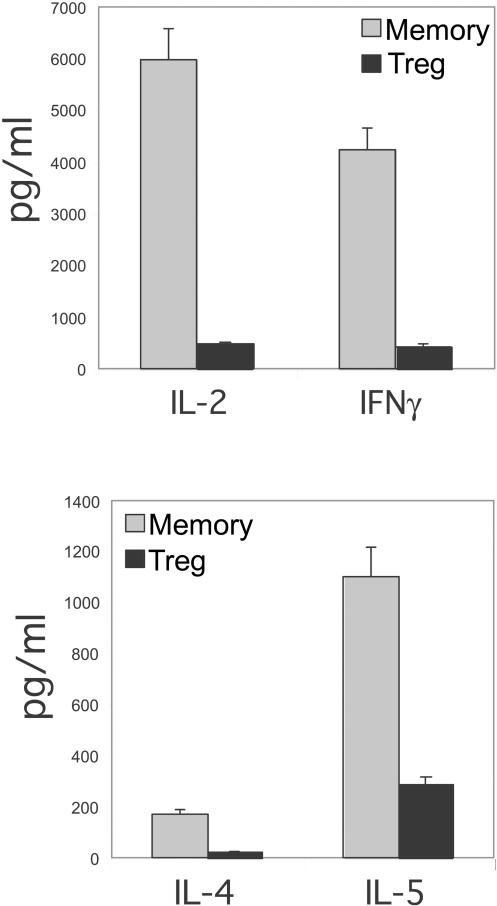
Cytokine Secretion by Treg T-cells Sorted Treg and memory T-cells were activated using plate-bound anti-CD3 (3 μg/ml) and soluble anti-CD28 (1 μg/ml) antibodies. Supernatants were collected 18–24 h postactivation and analyzed for cytokines using the CBA assay. Results are representative of cytokine secretion from Treg and memory T-cells from three different donors.

**Figure 3:**
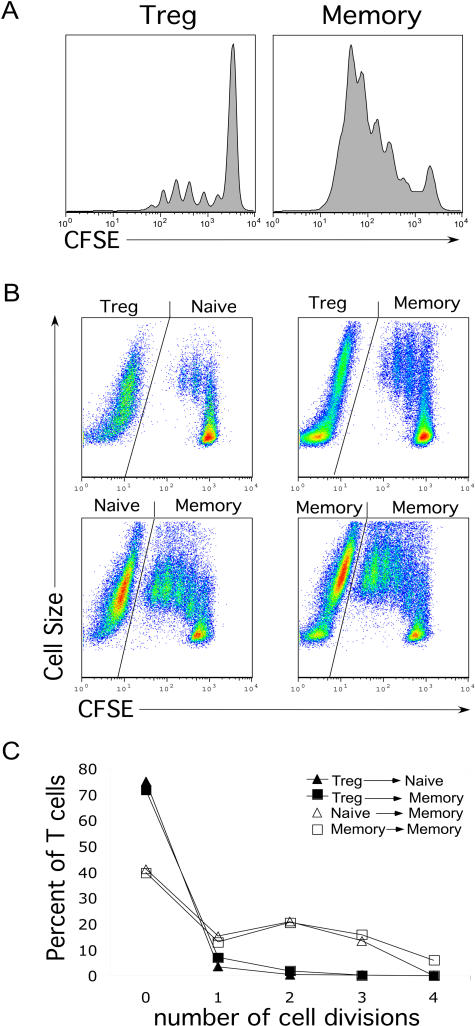
Proliferation and Suppressive Capacity of Treg Cells (A) Sorted Treg and memory T-cells were labeled with CFSE and then activated through suboptimal anti-CD3 (100 ng/ml) and anti-CD28 (1 μg/ml) antibodies. Day 6 postactivation, cells were fixed and CFSE expression was analyzed by flow cytometry. (B) Resting naïve or memory CD4^+^ T-cells (1.5 × 10^5^ T-cells) were labeled with CFSE and cocultured with either unlabeled purified Treg, naïve, or memory T-cells at 1:1 ratio in 96-well plates coated with suboptimal anti-CD3 (100 ng/ml) and anti-CD28 (1 μg/ml) antibodies. At day 4 postactivation, cells were fixed and analyzed for CFSE expression and cell size by flow cytometry. (C) Regions were set based on 2-fold reduction in CFSE mean intensity of naïve or memory T-cells as gated on (B), and plotted as number of cell divisions. Results represent three separate experiments from three different donors.

A low proliferative response and reduced IL-2 secretion are characteristics of Treg cells (Asano et al. 1996; Takahashi et al. 1998; Thornton and Shevach 1998; Baecher-Allan et al. 2001; Dieckmann et al. 2001; Jonuleit et al. 2001; Levings et al. 2001; Ng et al. 2001; Taams et al. 2001). To analyze their capacity to proliferate and secrete IL-2 upon TCR triggering, Treg and memory T-cells were sorted into highly purified populations by flow cytometry. Purified cells were then labeled with carboxy-fluorescein diacetate succinimidyl ester (CFSE) to monitor cell division in a quantitative manner and stimulated through the TCR using plate-bound anti-CD3 and soluble anti-CD28 antibodies. The secretion of IL-2 by TCR-stimulated Treg cells was about 10-fold lower as compared to memory T-cells (Figure 2). Treg cells also secreted lower levels of IL-4, IL-5, and IFNγ as compared to memory T-cells (Figure 2). The CFSE-labeled cells were analyzed 6 d after stimulation. Treg cells exhibited little proliferation, whereas most of the memory T-cells had divided four to five times (Figure 3A). In order to demonstrate that purified Treg cells also displayed suppressive activity, both naïve and CD25^low/neg^ memory CD4^+^ T-cells were labeled with CFSE and stimulated under suboptimal T-cell activation conditions in the presence of unlabeled purified autologous Treg, naïve, or memory T-cells. Coculture with Treg cells significantly slowed the proliferation of TCR-stimulated resting naïve and memory CD4^+^ T-cells as compared to the coculture with either unlabeled naïve or memory T-cells (Figure 3B and Figure3C). Taken together, these results confirm that human Treg cells are part of the CD4^+^CD25^hi^ subset of T-cells.

### Treg Cells Are Highly Susceptible to HIV Infection

**Figure 4:**
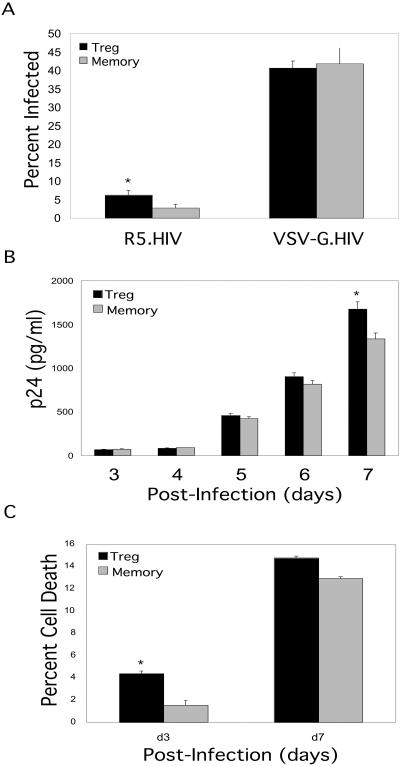
HIV Infection of Treg Cells (A) Sorted Treg and memory T-cells were activated using plate-bound anti-CD3 (3 μg/ml) and soluble anti-CD28 (1 μg/ml) antibodies and concurrently infected with R5.HIV or VSV-G.HIV at a MOI of 5 (based on prior virus titration using Hut78/CCR5 cells). The percentage of infected cells was determined by GFP expression at 3 d postinfection by flow cytometry. (B) Supernatants from Treg and memory T-cells infected with R5.HIV cultures were collected at different time points and HIV p24 levels were measured by ELISA. (C) Treg cell death was assessed by analyzing infected cells at days 3 and 7 postinfection based on forward- and side-scatter analysis and in some experiments using propidium iodine staining analysis with flow cytometry. All of the infection results are representative of one out of five separate experiments with reproducible results. Statistical significance was determined using the Student's two-tailed *t* test. * *p* < 0.05.

The ability to obtain a pure population of functional human Treg cells provides an excellent model to study their role in HIV pathogenesis. To determine whether Treg cells were susceptible to HIV infection, purified Treg cells were first activated through the TCR and were infected with either replication-competent HIV, which uses CCR5 as a coreceptor (R5.HIV), or replication-defective viruses pseudotyped with vesicular stomatitis virus glycoprotein (VSV-G.HIV) that encode green fluorescent protein (GFP) as a marker of infection (Motsinger et al. 2002). Treg and memory T-cells challenged with VSV-G.HIV resulted in an equivalent infection rate, while in some experiments R5.HIV resulted in about a 2-fold higher infection rate of the Treg cells as compared to the memory T-cells (Figure 4A). To determine the level of HIV replication in the Treg cells as compared to activated memory T-cells, both subsets were infected with R5.HIV for 2 d and washed to remove input virus. Supernatants were collected from the infected cultures daily and p24 levels were quantified by enzyme-linked immunosorbent assay (ELISA). HIV replicated in Treg cells as efficiently as in memory T-cells (Figure 4B). To assess whether the virus produced by Treg cells was infectious, supernatants from infected cells were added to Hut78/CCR5 cells, which are highly susceptible to HIV infection, and the titer of infectious virus was determined by GFP expression. Treg cells produced levels of infectious virus similar to those of the memory T subset (data not shown). The viability of infected cultures was also determined at days 3 and 7 to determine if HIV infection killed Treg cells. Indeed, infection with replication-competent HIV was highly cytotoxic to both Treg and memory T-cells (Figure 4C). We conclude that Treg cells are highly susceptible to HIV infection and are killed by viral replication.

### Genetic Reprogramming of Conventional Human T-cells into Treg Cells by Ectopic Expression of FoxP3

Treg cells constitute less than 1%–2% of total human T-cells (see Baecher-Allan et al. 2001; see Figure 1). Although we could purify several hundred thousand Treg cells from 300 ml of blood as described below, isolation of sufficient numbers of Treg cells is clearly an obstacle to studying their function and susceptibility to HIV infection. Recently, a transcription factor called FOXP3 was shown to program murine T-cells into a Treg subset (Fontenot et al. 2003; Hori et al. 2003; Khattri et al. 2003). Therefore we hypothesized that ectopic expression of FoxP3 in naïve T-cells could facilitate the generation of large numbers of human Treg cells. Accordingly, we subcloned *FoxP3* cDNA into a HIV-derived vector (HDV) that encodes murine CD24 (mCD24) as a marker (Sundrud et al. 2003). CD4^+^ T-cells were purified from both neonatal cord blood (CB) and adult blood (AB), activated through the TCR and transduced with FoxP3-expressing HDV (HDV.FoxP3) or control HDV as described previously (Sundrud et al. 2003). Expression of *FoxP3* mRNA in transduced cells was confirmed by real-time PCR analysis and was found to be about 50- to 100-fold higher in FoxP3 transduced primary CD4^+^ T-cells as compared to control HDV-transduced cells (data not shown).

**Figure 5:**
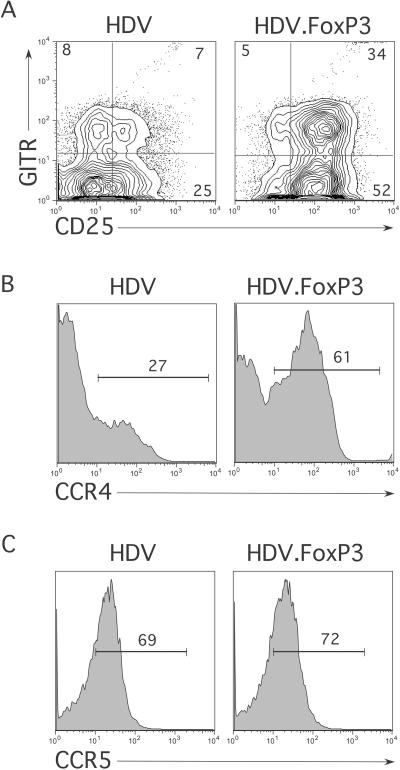
Phenotype of FoxP3 Transduced T-cells Purified CB naïve CD4^+^ T-cells were activated through the TCR and transduced with either HDV.FoxP3 or HDV. Cells were expanded for 14 d in IL-2-containing medium and stained with (A) anti-mCD24, anti-GITR, and anti-CD25, (B) anti-mCD24 and anti-CCR4, or (C) anti-mCD24 and anti-CCR5 antibodies. Gates were set on the mCD24-positive population (transduced), and expression of surface molecule was analyzed. The expression of these markers in the CD24-negative portion of both cultures was identical (data not shown). These results are representative of T-cells isolated from five different donors and transduced independently.

**Figure 6:**
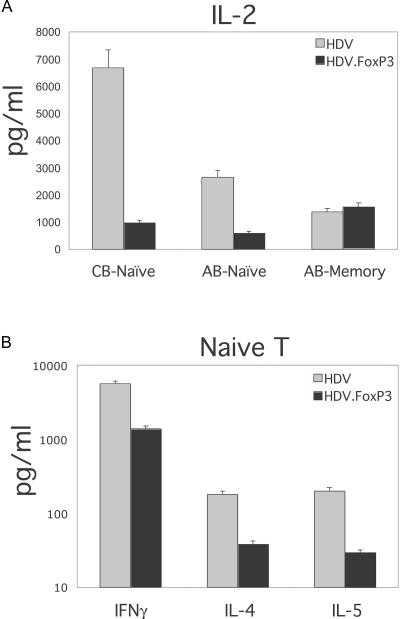
Cytokine Production by FoxP3-Transduced T-cells CD4^+^ naïve T-cells isolated from CB (CB-naïve) and AB (AB-naïve) and memory T-cells from AB (AB-memory) were transduced with HDV or HDV.FoxP3 as described in Figure 5. Transduced T-cells were purified through magnetic sorting of mCD24^+^ cells and activated using plate-bound anti-CD3 and soluble anti-CD28 antibodies. Supernatants were collected at 18–24 h postactivation and analyzed for (A) IL-2 production or (B) IFNγ, IL-4, and IL-5 production from HDV or HDV.FoxP3-tranduced naïve T-cells, using CBA assay. The results represent five separate experiments from different donors with similar relative differences in cytokine production.

**Figure 7:**
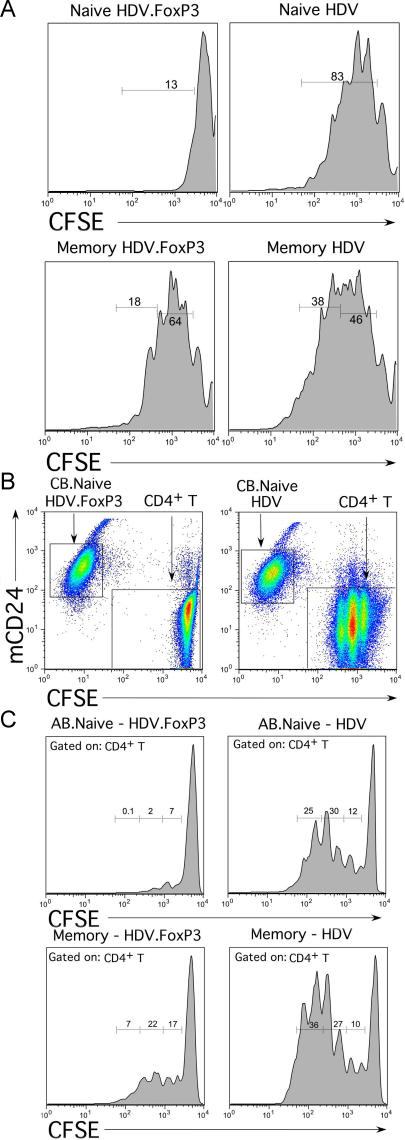
Proliferation and Suppression by FoxP3-Expressing Cells (A) Purified CD4^+^ naïve and memory T-cells were transduced with either HDV.FoxP3 or HDV as described. The transduced T-cells were labeled with CFSE and activated with anti-CD3 (100 ng/ml) and anti-CD28 (1 μg/ml) antibodies. Day 6 postactivation, cells were fixed and analyzed for CFSE expression by flow cytometry. (B) Resting CD4^+^ T-cells (1.5 × 10^5^) were labeled with CFSE and cocultured at 1:1 with either unlabeled sorted HDV.FoxP3-expressing or HDV-transduced CB naïve T-cells and activated with anti-CD3 (100 ng/ml) and anti-CD28 (1 μg/ml ) antibodies. At 4 d postactivation, cells were stained with mCD24-PE as a marker for infection. (C) Naïve and memory T-cells isolated from adult blood were transduced with HDV.FoxP3 or HDV. A coculture suppression experiment was set up with resting purified autologous CD4^+^ T-cells as described above. Region was set on mCD24 negative CFSE^+^ cells (target resting CD4^+^ T-cells) as shown in (B), and CFSE expression was analyzed 6 d poststimulation by flow cytometry. The results are representative of three separate experiments.

FoxP3-transduced and control cells were expanded for 14 d in IL-2-containing medium. Cells were then stained for mCD24 and also for CD25, GITR, and CCR4 markers that are expressed at higher levels on naturally occurring Treg cells (see Figure 1). Naïve T-cells ectopically expressing FoxP3 displayed higher levels of CD25, GITR (Figure 5A), and CCR4 (Figure 5B) as compared to control transduced T-cells. FoxP3-transduced memory T-cells displayed much less upregulation of these markers (data not shown). However, while the majority of these transduced T-cells also expressed CCR5, its expression levels on FoxP3-transduced and control T-cells were similar (Figure 5C). To determine whether FoxP3-transduced T-cells display functional properties such as hyporesponsiveness to TCR triggering, similar to freshly isolated Treg cells, transduced cells were purified by sorting mCD24^+^ cells as described previously (Sundrud et al. 2003). Equal numbers of purified cells were then stimulated using plate-bound anti-CD3 and soluble anti-CD28 antibodies, and cytokine secretion was monitored. Secretion of IL-2 from both CB and AB naïve T-cells was reduced between 8- and 10-fold in FoxP3-expressing cells as compared to control cultures (Figure 6A). Secretion of IL-4, IL-5, and IFNγ was also reduced in FoxP3-expressing naïve T-cells (Figure 6B). In contrast, FoxP3-transduced memory T-cells secreted similar levels of IL-2 as compared to cells transduced with HDV alone (Figure 6A). To assess the proliferative capacity of FoxP3-expressing cells, transduced cells were labeled with CFSE and stimulated through the TCR. After 4 d of activation, very few FoxP3-transduced naïve T-cells had divided as compared to control lines (Figure 7A). Although, FoxP3-expressing memory T-cells also divided fewer times as compared to HDV-transduced cells, the effect of FoxP3 was greatly diminished in this subset (Figure 7A).

The key characteristic of Treg cells is suppression of conventional T-cells activated through the TCR (Takahashi et al. 1998; Thornton and Shevach 1998). Thus, purified resting CD4^+^ T-cells were labeled with CFSE and cocultured with unlabeled naïve or memory T-cells that were transduced either with HDV.FoxP3 or HDV and then stimulated through the TCR as described for freshly isolated Treg cells (see Figure 3). FoxP3-expressing naïve T-cells, both from CB or AB, completely suppressed proliferation of target resting CD4^+^ T-cells (Figure 7B and Figure7C). A significant but lower level of suppression was apparent with memory T-cells transduced with FoxP3 (Figure 7C). We conclude that ectopic expression of FoxP3 in naïve human T-cells recapitulates key phenotypic and functional features of naturally occurring Treg cells.

### T-cells Ectopically Expressing FoxP3 Are More Susceptible to HIV Infection

**Figure 8:**
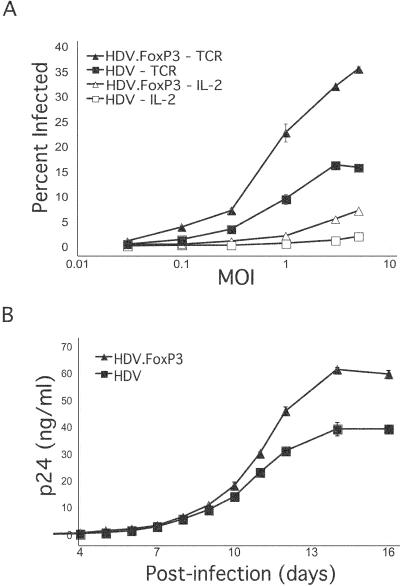
HIV Infection of FoxP3-Expressing T-cells (A) HDV.FoxP3 and HDV-transduced T-cells were activated using plate-bound anti-CD3 (100 ng/ml) and soluble anti-CD28 (1 μg/ml) antibodies or maintained in IL-2-containing medium. Cells were concurrently infected at different MOI of VSV-G.HIV, and infection was determined by GFP expression at 72 h postinfection by flow cytometry. (B) Supernatants were collected at different time points from R5.HIV-infected HDV.FoxP3-expressing or HDV-transduced cell cultures, and HIV p24 levels were measured by ELISA. The percentages of infected cells at days 3, 9, and 16 for HDV.FoxP3 were 2, 10, and 26, and for HDV were 0.8, 10, and 18, respectively.

We next determined the susceptibility of FoxP3-expressing cells to HIV infection. FoxP3-transduced cells were purified by flow cytometry sorting based on mCD24 expression and activated through their TCR or cultured in IL-2-containing medium. Subsequently, activated cells were challenged with either VSV-G.HIV or R5.HIV. Remarkably, FoxP3-expressing cells were infected at a level about 2- to 3-fold higher than control cells at different concentrations of the virus, in both activated and nonactivated conditions (Figure 8A). FoxP3-expressing cells stimulated at suboptimal levels of anti-CD3 antibody also displayed a very similar enhancement of infection compared to HDV-transduced cells (data not shown).

We next analyzed the level of HIV replication and cell death in FoxP3-expressing cells as compared to HDV-transduced T-cells. Activated FoxP3-expressing and control cells were infected with R5.HIV, and culture supernatants were collected daily from day 3 postinfection. FoxP3-expressing cells showed increased HIV-infection and replication (Figure 8B). Infectivity of virus produced by FoxP3-expressing cells, as assessed on Hut78/CCR5 cells, as well as cell death in these cultures, was also proportionately higher (data not shown). These findings demonstrate that the expression of FoxP3 renders CD4^+^ primary T-cells more susceptible to HIV infection.

### HIV-Infected Patients Have Greatly Decreased Levels of FoxP3-Expressing CD4^+^CD25^hi^ T-cells

**Figure 9:**
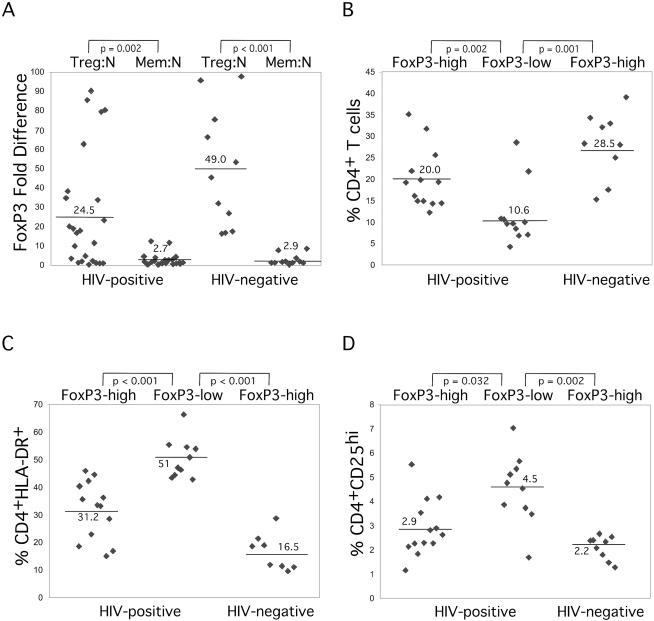
FoxP3 Expression in Purified CD4^+^CD25^hi^ (Treg), Naïve, and Memory T-cells from HIV-Infected and Healthy Individuals (A) RNA was isolated from sorted Treg, naïve, and memory T-cells from HIV-positive (*n* = 24) and HIV-negative (*n* = 11) subjects, followed by cDNA synthesis. *FoxP3* expression was quantified by TaqMan real-time PCR. The *FoxP3*-fold difference expression was calculated for CD4^+^CD25^hi^ (Treg) versus naïve (N), and memory (Mem) versus naïve (N) T-cells. Treg cells sorted from HIV-positive subjects were further subdivided into two groups based on *FoxP3* expression of Treg compared to naïve T-cells (FoxP3-high, *n* = 13, FoxP3 difference >10-fold; FoxP3-low, *n* = 11, FoxP3 difference <10-fold; HIV-negative, *n* = 9). These groups were stained with anti-CD3, anti-CD4, anti-CD45RO, anti-CD25, and anti-HLA-DR and analyzed by flow cytometry for (B) CD4^+^ T-cell percentage, (C) activated T-cell percentage (CD4^+^HLA-DR^+^), and (D) CD4^+^CD25^hi^ percentage. Horizontal lines identify means. Statistical significance between groups was determined by Mann–Whitney *U* test and shown on top of each figure.

Our findings that Treg cells are highly susceptible to HIV infection prompted us to determine if this subset was disturbed within HIV-infected individuals. However, a major difficulty in such analysis is that many of the cell surface markers that define Treg cells are also expressed on activated T-cells (CD25, HLA-DR, GITR). Because a portion of HIV-positive individuals contain high levels of activated T-cells, simple cell surface analysis would not be sufficiently reliable to quantify Treg cells in these donors. Therefore, we utilized FoxP3 expression as the most reliable marker that defines Treg cells. To accomplish this, we sorted CD4^+^CD25^hi^, naïve, and memory T-cells from 11 HIV-negative, healthy donors (median age, 31; 64% male) and 24 HIV-infected individuals (median age, 38; 85% male; 88% receiving antiretroviral therapy). Total RNA was then isolated from each subset and *FoxP3* mRNA expression was quantified using real-time PCR analysis. In order to normalize for experimental variability, *FoxP3* expression of the CD4^+^CD25^hi^ cells was normalized to *GAPDH* levels from the same samples and compared to the naïve T-cell subset isolated from the same donor. We found that within HIV-negative subjects there was on average a 49-fold higher level of expression of *FoxP3* in CD4^+^CD25^hi^ cells as compared to naïve T-cells (Figure 9A). The lowest FoxP3-expressor in the healthy subject group had 16-fold higher FoxP3 expression as compared to naïve T-cells from the same donor (Figure 9A). There was a similar increase in *FoxP3* expression as compared to memory T-cells (data not shown). In HIV-positive subjects *FoxP3* expression was only increased a mean of 25-fold in CD4^+^CD25^hi^ cells. In contrast to healthy donors, we also observed that in about half of the HIV-positive subjects, CD4^+^CD25^hi^ cells expressed very low to undetectable levels of *FoxP3* (Figure 9A). *FoxP3* expression in memory T-cells was similar in HIV-positive and HIV-negative subjects (Figure 9A).

Progressive HIV disease is associated with decreased CD4^+^ T-cell percentages and increased levels of activated T-cells. We hypothesized that this hyperactivation may be due to a loss of Treg cells. Therefore, to further evaluate relationships between FoxP3 expression and these parameters in HIV-infected individuals, samples were divided into low FoxP3 expressors (less than 10-fold higher expression in CD4^+^CD25^hi^ T-cells compared to naïve T-cells, designated FoxP3-low) versus high FoxP3 expressors (greater than 10-fold higher expression compared to naïve T-cells, designated FoxP3-high). HIV-positive subjects with a FoxP3-low profile had significantly lower CD4^+^ T-cell percentages, while FoxP3-high HIV-positive subjects had CD4^+^ T-cell percentages comparable to HIV-seronegative subjects (Figure 9B). Similarly, HIV-positive FoxP3-low subjects had significantly greater activated CD4^+^T-cells (CD4^+^HLA-DR^+^) than either HIV-positive subjects with FoxP3-high profiles or HIV-seronegative subjects (Figure 9C). Interestingly, the CD4^+^CD25^hi^ T-cells are also significantly increased in FoxP3-low expressors as compared to HIV-negative or HIV-positive FoxP3-high expressors (Figure 9D). These differences are most likely due to recently activated T-cells that also express high levels of CD25, as shown by higher HLA-DR expression on T-cells from the same subset of subjects (Figure 9C). Among HIV-positive subjects there was no significant association between FoxP3 expression and plasma HIV-1 RNA concentration, age, race, sex, or whether the subject was receiving antiretroviral therapy (*P* > 0.05 for each comparison). These findings demonstrate that a decrease in Treg cells is associated with HIV disease progression and suggest that loss of Treg cells may contribute to increased T-cell hyperactivation.

## Discussion

In this study we demonstrated that human Treg cells are highly susceptible to HIV infection and that ectopic expression of FoxP3 genetically reprograms conventional naïve T-cells, phenotypically and functionally, into Treg cells. Remarkably, overexpression of FoxP3 also greatly enhances the susceptibility of activated T-cells to HIV infection. Although Treg cells constitute a small subset of the total T-cells in humans (less than 1%–2%) and thus may not be a significant target population for HIV, they appear to have very potent suppressive activity against activation of T-cells (Takahashi et al. 1998; Thornton and Shevach 1998; Baecher-Allan et al. 2001; Curotto de Lafaille and Lafaille 2002). Here we demonstrate that FoxP3-expressing CD4^+^CD25^hi^ T-cells are greatly decreased in a portion of HIV-infected individuals with low CD4 and high activated T-cells, suggesting a loss of Treg cells. We therefore propose that infection and disruption of Treg cells during HIV infection could have a major influence on T-cell homeostasis and immune regulation.

Similar to previous reports, our findings demonstrate that human Treg cells appear to be enriched within the CD25^hi^ subset of CD4^+^ T-cells. However, it is not clear if Treg cells are the only population represented in the CD25^hi^ subset since they share this phenotype with recently activated T-cells. Indeed, a portion of purified CD25^hi^ cells proliferated and their suppressive function was less efficient as compared to FoxP3-expressing cells (see Figure 3). Because the purification of Treg cells is rather arbitrary (brightest 1%–2% of antibody-stained CD25^+^ memory T-cells), it is conceivable that there is sizable contamination of non-Treg cells in these sorted preparations. In addition, the differences seen in the infection susceptibility of Tregs as compared to FoxP3-expressing cells may also be partly due to presence of non-Treg activated T-cells within the purified cells. Identification of the Treg subset in disease conditions with chronic T-cell activation, such as HIV, is even more problematic because a large proportion of CD4^+^CD25^hi^ cells possibly represent recently activated T-cells. Availability of large numbers of genetically reprogrammed Treg cells should facilitate the identification of novel markers that can reliably detect human Treg cells.

Our results clearly demonstrate that, similar to the mouse system, ectopic expression of FoxP3 is sufficient to recapitulate all of the characteristics of Treg cells, including lower cytokine secretion, higher expression of CD25 and GITR, and their suppressive functions. This system has allowed us to generate large numbers of Treg cells, which will be invaluable in characterizing their suppressive function as well as mechanisms of enhanced HIV susceptibility. The ability to genetically manipulate primary T-cells to reprogram them into the Treg phenotype also could have profound implications for preventing graft-versus-host disease, a serious clinical condition that can be manifested following hematopoietic cell transplantation (Hoffmann et al. 2002; Taylor et al. 2002).

It is interesting to note that naïve T-cells are more prone to reprogramming into a Treg phenotype than memory T-cells are. This loss in flexibility of reprogramming with a master transcription factor is reminiscent of Th1- and Th2-type T-cell reprogramming with ectopic expression of lineage-specific transcription factors T-bet and GATA-3, respectively (Sundrud et al. 2003). The loss of flexibility in genetic modification of effector/memory T-cells could reflect heritable epigenetic changes at effector gene loci that might otherwise be responsive to FoxP3-mediated transcription. A recent study supports this hypothesis by demonstrating that lineage-committed human memory cells failed to modify their histone acetylation patterns of cytokine genes, unlike naïve T-cells, to differentiate into Th1- or Th2-type cells (Messi et al. 2003).

The cause of progressive depletion of CD4^+^ cells and the reason for high T-cell activation or turnover during HIV infection remains controversial (Hazenberg et al. 2000; Grossman et al. 2002). It is thought that HIV-mediated destruction of CD4^+^ T-cells results in decline of this subset and that to maintain homeostasis the immune system attempts to replenish this subset, resulting in a massive turnover of T-cells (Ho et al. 1995; Wei et al. 1995; Perelson et al. 1996; Mohri et al. 1998, 2001). This excessive turnover rate eventually compromises proper function of homeostatic responses. Alternatively, T-cell depletion could result from disrupted thymic and peripheral homeostatic mechanisms by virus-induced generalized T-cell activation and gradual wasting of T-cell supplies, eventually leading to T-cell depletion (Hazenberg et al. 2000; Grossman et al. 2002).

Indeed, several mechanisms control unwanted activation of T-cells, including thymic deletion of autoreactive T-cells and induction of anergy in the periphery. In addition to these passive mechanisms, recent evidence clearly demonstrates that Treg cells exert an active suppression of T-cell activation. We postulate that the high susceptibility of Treg cells to HIV in vivo, as demonstrated by our in vitro studies, could result in gradual elimination of this subset. This Treg cell decline during HIV infection would in turn reduce active suppression of conventional T-cells and, hence, contribute to hyperactivation of T-cells. Our analyses of peripheral blood T-cells from HIV-infected subjects support this hypothesis that loss of Treg cells during HIV infection contributes to HIV disease progression. Indeed, we found that in a subset of HIV-infected subjects, the CD4^+^CD25^hi^ T-cell subset had greatly reduced FoxP3 expression, suggesting that these cells represent recently activated T-cells rather then Treg cells. The presence of a higher percent of activated T-cells in this FoxP3-low profile supports this conjecture. The HIV-infected subjects with lower levels of FoxP3^+^ T-cells also contained a lower percentage of CD4^+^ T-cells. It is conceivable that the loss of Treg cells may be a correlative factor for disease progression; however, more detailed prospective studies will be required to address this important implication of our findings.

Our findings show a 2- to 3-fold enhancement of HIV infection in FoxP3-expressing T-cells. While freshly purified Treg and memory T-cells are similar in their susceptibility to HIV infection, activated effector T-cells become gradually more resistant to infection (unpublished results). Indeed, preactivated T-cells require reactivation to render them susceptible to infection after about 1–2 weeks in culture (unpublished results). We do not yet know the mechanisms by which FoxP3 renders activated T-cells more susceptible to infection; however, two possibilities can be considered: (1) FoxP3 may be overcoming innate resistance factor(s) that accumulate in activated T-cells that block HIV infection, or (2) FoxP3 expression may be inducing critical host factors that are required for efficient completion of the HIV life cycle in primary T-cells. It is important to note that since FoxP3 expression enhances VSV-G.HIV single-round infections, it likely affects an early, postentry step in viral replication. Determining the mechanisms by which FoxP3 enhances HIV infection could reveal host factors involved in this process.

In summary, our results indicate that both naturally occurring and genetically reprogrammed Treg cells are susceptible to HIV infection and that ectopic FoxP3 expression greatly increases the susceptibility of T-cells to HIV infection. Our finding that FoxP3-expressing CD4^+^CD25^+^ T-cells are greatly reduced in HIV patients with low CD4^+^ T-cell percentages and increased T-cell activation suggests that loss of Treg cells may contribute to HIV disease progression. Further prospective studies will be required to unravel the role of this important subset in HIV infection. The ability to genetically reprogram conventional human T-cells to generate Treg cells will also lead the way to identifying unique markers expressed on this population in order to further investigate their status in HIV-infected individuals. Moreover, understanding how FoxP3 enhances HIV infection and programs T-cells into the Treg subset could help in the identification of novel host factors that mediate HIV infection in primary T-cells and decoding the molecular mechanisms by which Treg cells mediate their suppressive function.

## Materials and Methods

### 

#### Study subjects and statistical analysis

Healthy subjects (*n* = 11) were adults who were HIV-negative and with no history of chronic viral infections such as Hepatitis B or C. Blood samples from adults with HIV infection (*n* = 24) were obtained during routine primary care visits at the Comprehensive Care Center, Vanderbilt University Medical School, Nashville, Tennessee, United States. There were no selection criteria based on race or sex. All subjects provided written consent, and the study was approved by the Vanderbilt Institutional Review Board. Continuous variables were compared by a Mann–Whitney *U* test, and categorical variables by an χ^2^ test. All significance levels were based on two-tailed tests. Statistical analyses were performed using SPSS, version 12.0 (SPSS, Chicago, Illinois, United States).

#### Cell isolation and culture

Peripheral blood mononuclear cells (PBMCs) were separated from buffy coats of healthy and HIV-positive donors through Ficoll–Hypaque separation (Pharmacia-LKB Technology, Uppsala, Sweden). Resting CD4^+^ T-cells were purified as previously described (Unutmaz et al. 1999). This purification protocol typically resulted in 99.5% purity of positively selected cells, as determined by postpurification fluorescence-activated cell sorting (FACS) analysis. To isolate Treg cells, PBMCs, or purified CD4^+^ cells, were stained with CD45RO, CD25, CD4, and HLA-DR antibodies (BD Biosciences Pharmingen, San Diego, California, United States), and CD4^+^CD45RO^+^CD25^hi^ and CD4^+^CD45RO^−^CD25^low/neg^ cells were sorted using flow cytometry (FACS Aria; BD Biosciences Pharmingen). For some experiments, adult CD4^+^ T-cells were sorted into CD45RO^+^ (memory) and CD45RO^−^ (naïve) T-cells with anti-CD45RO conjugated magnetic beads (Miltenyi Biotec, Bergisch Gladbach, Germany) using AutoMACS (Miltenyi Biotec). Purified resting T-cells were activated by cross-linking with plate-bound anti-CD3 antibody (OKT-3; American Type Culture Collection, Manassas, Virginia, United States) and soluble anti-CD28 antibody (1 μg/ml, BD Biosciences Pharmingen). The plates were first coated with goat antimouse IgG (10 μg/ml, CalTag Laboratories, Burlingame, California, United States) followed by either 3 μg/ml anti-CD3 for optimal TCR stimulation or 100 ng/ml anti-CD3 for suboptimal stimulation. The culture medium used in all experiments was RPMI (Life Technologies, Carlsbad, California, United States) and was prepared as previously described (Motsinger et al. 2002). All cytokines were purchased from R & D Systems (Minneapolis, Minnesota, United States). Monocyte-derived dendritic cells were generated as previously described (Motsinger et al. 2002). Superantigen, staphylococcal enterotoxin B (Sigma, St. Louis, Missouri, United States) was used to stimulate resting T-cells in the presence of dendritic cells (Motsinger et al. 2002).

#### Virus production and infections

VSV-G.HIV particles were generated as previously described (Unutmaz et al. 1999). R5-HIV was prepared similarly by transfecting 293T-cells with HIV that encodes R5-tropic (BAL) envelope and enhanced GFP (Clontech, Palo Alto, California, United States) in place of the *nef* gene as previously described (Unutmaz et al. 1999). Typically viral titers ranged from 1–5 × 10^6^ ifu/ml for replication-competent viruses and 10–30 × 10^6^ for VSV-G.HIV. T-cells were infected at varying multiplicities of infection (MOI), and infection was quantified by GFP expression using flow cytometry. In some experiments, cells inoculated with virus were centrifuged for 1 h at 2,000 rpm to enhance infectivity, as described by O'Doherty et al. (2000). Viral replication in T-cell cultures was determined by measuring p24 levels within supernatants by an ELISA (Motsinger et al. 2003), and infectious virus production by infected T-cells was determined by culturing Hut78 cells expressing CCR5 (Hut78/CCR5) (Wu et al. 2002) in infected T-cell supernatants.

#### CFSE labeling

Cell division was measured by labeling the T-cells with CFSE (Molecular Probes, Eugene, Oregon, United States). Purified cells were first washed and resuspended in PBS. While vortexing the cells, CFSE was added at a final concentration of 5 μM. The mixture was vortexed for an additional 15 s and incubated at 37 °C for 3 min. Labeling was quenched by addition of 50% fetal calf serum in PBS. Cells were washed one more time with 50% serum PBS, followed by two washes with RPMI-supplemented medium. All CFSE labeling and culturing were performed under dark conditions.

#### FACS analysis and cytokine assay

T-cells were stained with the relevant antibody on ice for 30 min in PBS buffer containing 2% fetal calf serum and 0.1% sodium azide. Cells were then washed twice, fixed with 1% paraformaldehyde, and analyzed with a FACSCalibur four-color cytometer. Live cells were gated based on forward- and side-scatter properties, and analysis was performed using FlowJo software (Tree Star, Ashland, Oregon, United States). The following antihuman antibodies were used for staining: CD3, CD4, CD45RO, CD45RA, CD25, GITR, HLA-DR, CCR5, CCR4, CCR7, CXCR4, CXCR3, and antimouse CD24, all obtained from PharMingen (San Diego, California, United States). Cytokines (IL-2, IL-4, IL-5, and IFNγ) in the supernatants were assayed using a commercially available cytometric bead array (CBA) (BD Biosciences Pharmingen) (Cook et al. 2001) and analyzed using CBA 6-bead analysis software (BD Biosciences Pharmingen).

#### Cloning of human FoxP3

RNA was isolated from activated human T-cells using an RNeasy kit (Qiagen, Valencia, California, United States). To synthesize cDNA 100 ng RNA was used (Superscript II Reverse Transcriptase; Invitrogen, Carlsbad, California, United States). *FoxP3* was PCR amplified from T-cell cDNA with the following primers: *FoxP3* forward, 5′-AGATATCCCAGCCATGCCCAACCCCAGGCCTGGCAAG-3′; *FoxP3* reverse, 5′-TCAGGGGCCAGGTGTAGGGTTGGAACACCT-3′. The forward primer included an EcoRV restriction site to facilitate cloning. The *FoxP3* PCR product was subcloned into a TOPO shuttle vector (pcDNA3.1/CT-GFP-TOPO; Invitrogen). Following an EcoRV digest, FoxP3 was ligated into an HDV-encoding mCD24 down stream of an internal ribosome entry site (Sundrud et al. 2003). The FoxP3 coding sequence was confirmed by DNA sequencing.

#### Real-time PCR protocol

RNA was extracted, as described above, from cells transduced with HDV or HDV.FoxP3 or sorted Treg, naïve, and memory T-cells from HIV-positive and HIV-negative subjects. RNA (100 ng) was used to synthesize cDNA (as described above). Taqman Assays-on-Demand Gene Expression Primers (Applied Biosystems, Foster City, California, United States) were used in real-time PCR analyses: *GAPDH* primer mix assay ID Hs99999905_m1; *FoxP3* primer mix assay ID Hs00203958_m1.

Real-time PCR was performed using the ABI 7700 apparatus (PE Applied Biosystems, Weiterstadt, Germany). The reaction mixtures (20-μl total volume) contained 2 μl of serially diluted cDNA, 10 μl of Taqman Universal PCR Master Mix (PE Applied Biosystems), and 1 μl of either *FoxP3* or *GAPDH* primer mix. The reactions were amplified as follows: 50 °C for 2 min and 95 °C for 10 min, followed by 40 cycles of 95 °C for 1 min and 65 °C for 1 min. Expression of *FoxP3* was normalized to *GAPDH* expression in each sample.

## Supporting Information

### Accession Number

The GenBank (http://www.ncbi.nlm.nih.gov/) accession number for the gene *FoxP3* discussed in this paper is AF277993.

## Abbreviations

AB: adult blood
CB: cord blood
CBA: cytometric bead array
CFSE: carboxy-fluorescein diacetate succinimidyl ester
ELISA: enzyme-linked immunosorbent assay
FACS: fluorescence-activated cell sorting
GFP: green fluorescent protein
GITR: glucocorticoid-induced tumor necrosis factor receptor
HDV: HIV-derived vector
HDV.FoxP3: FoxP3-expressing HIV-derived vector
MOI: multiplicity of infection
R5.HIV: CCR5-tropic HIV
TCR: T-cell receptor
Treg cell: regulatory T-cell
VSV-G.HIV: HIV pseudotyped with vesicular stomatitis virus glycoprotein envelope
